# Supplementary material for: Ginsentide TP1 Protects Hypoxia-Induced Dysfunction and ER Stress-Linked Apoptosis
Source: Cells. 2023 May 16;12(10):1401. doi: 10.3390/cells12101401 (PMC10216702; doi:10.3390/cells12101401)
Supplement: Supplementary file 1 [file cells-12-01401-s001.zip › Supplementary Data S1.pdf]

Ginsentide TP1 Protects Hypoxia-Induced Dysfunction and ER Stress-Linked Apoptosis

Bamaprasad Dutta[1], Shining Loo[1, 2], Antony Kam[1, 3], Siu Kwan Sze[1, 4] and James P. Tam[1]*

1School of Biological Sciences, Nanyang Technological University, Singapore 637551, Singapore
2Academy of Pharmacy, Xi'an Jiaotong-Liverpool University, Suzhou 215123, China
3Department of Biological Sciences, Xi'an Jiaotong-Liverpool University, Suzhou 215123, China
4Department of Health Sciences, Brock University, Niagara Region, St. Catharines, ON L2S 3A1, Canada

***Correspondence:**
Prof James P Tam, PhD
School of Biological Sciences
Synthetic Enzymes and Natural Products Center
Nanyang Technological University,
60 Nanyang drive, Singapore 637551
Tel: (+65) 6316-2833
Email: jptam@ntu.edu.sg

Supplementary Figures

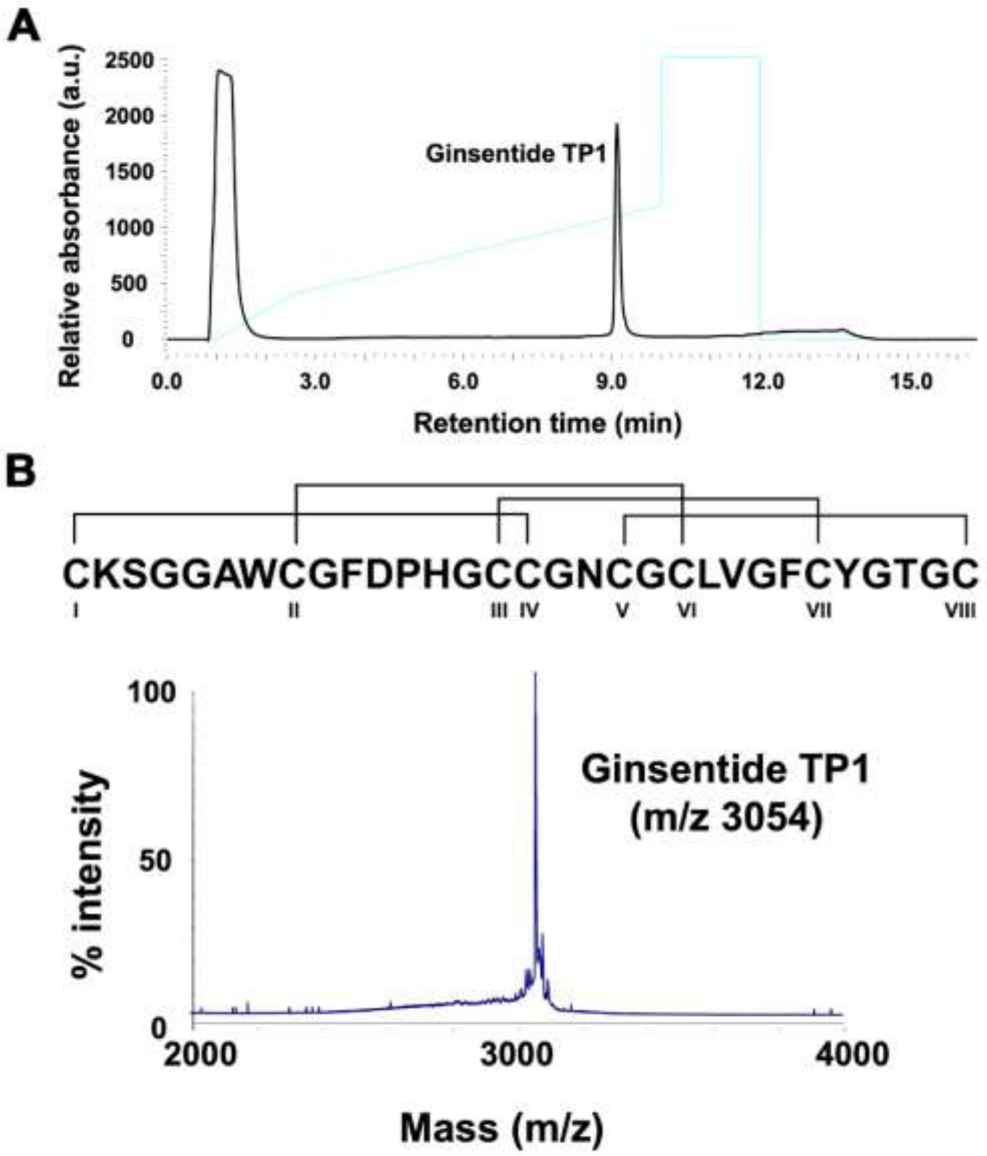

Figure S1: HPLC and mass spectrometry profiles of purified ginsentide TP1 extracted from *Panax ginseng* flowers. A) HPLC chromatogram of purified TP1. B) Mass spectrometry profile of purified TP1.

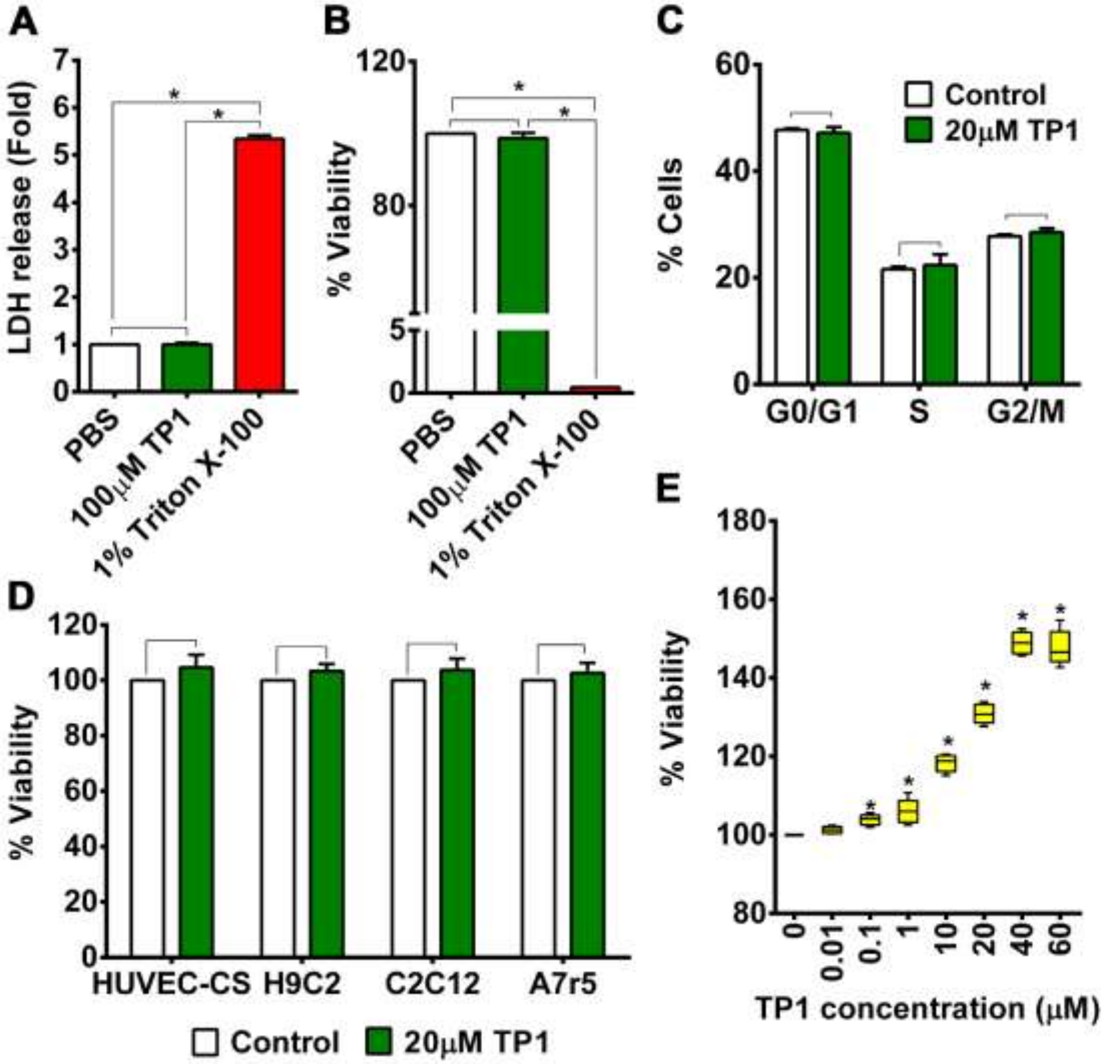

Figure S2: Toxicity and dose–response curve of ginsentide TP1. A, B) Effect of TP1 on cell viability/cytotoxicity and membrane integrity in HUVEC-CS cells. Precultured cells were treated with 100 µM TP1 for 24 h. PBS and 1% TritonX-100 were used as a vehicle, and positive (cell death) controls, respectively. Cytotoxicity and membrane-damaging effects were measured using an LDH release assay (A), and cell viability was assessed using an MTT assay (B). Relative quantifications are expressed as means ± standard deviations (SDs), and statistical significance was calculated using four biological replicates. *P < 0.05. C) Cell cycle analysis of 20 µM TP1–treated and vehicle control–treated HUVEC-SC cells. DNA content of the cells was measured using flow cytometry after propidium iodide staining. Data from cells in different cell cycle phases are expressed as means ± SDs, and statistics were calculated using triplicate biological replicates. D) Viability of different cells was measured using an MTT assay after treatment with 20 µM TP1 or vehicle control. Relative survival is expressed as mean values and statistical significance was calculated using three independent experimental replicates. E) Dose–response curve of TP1. HUVEC-CS cells were cultured and treated with different doses (0–60µM) of TP1 for 24 h in a hypoxic environment, and an MTT assay was used to assess cell viability. Data are means ± standard error of the mean of five independent experimental replicates. *P < 0.05.

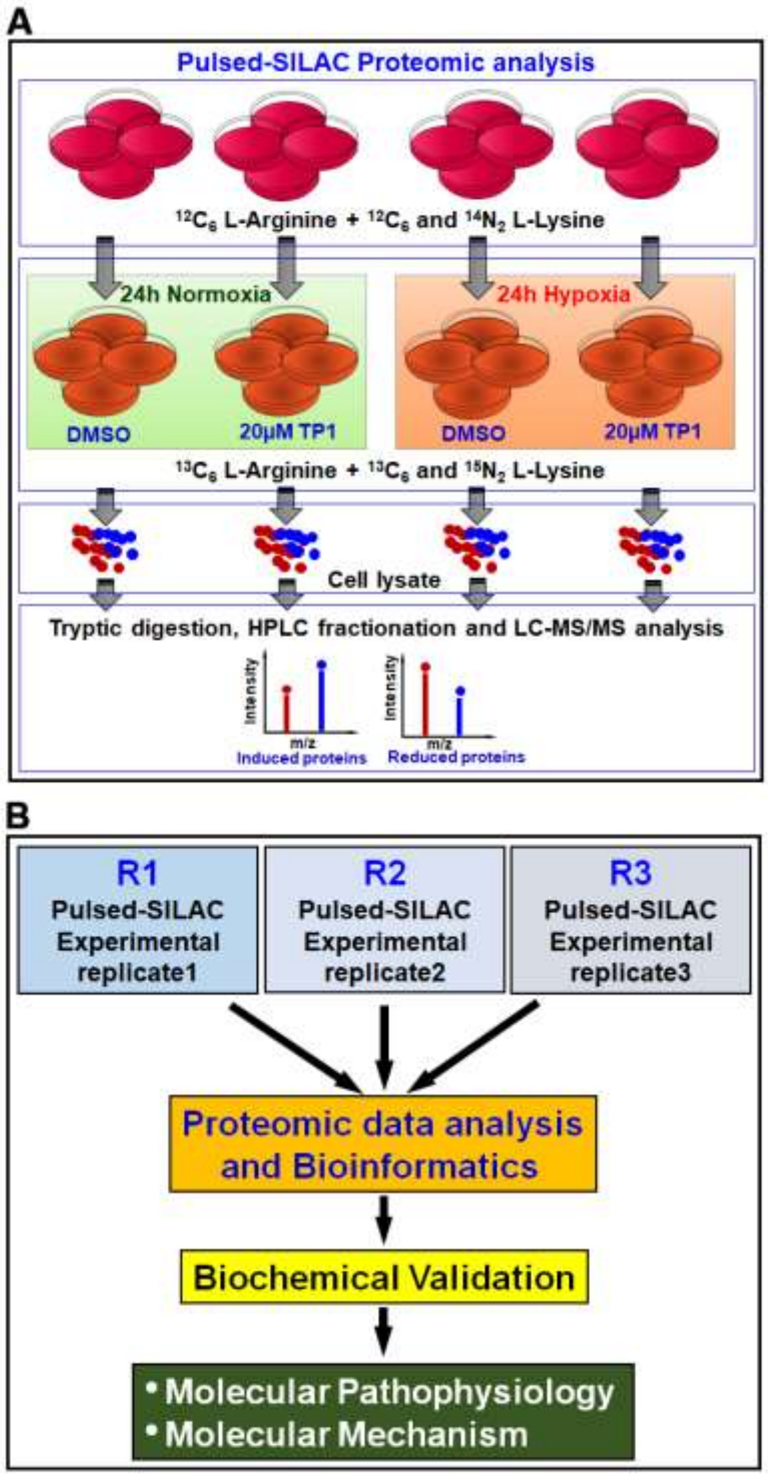

Figure S3: Schematic representation of the experimental workflow of pulsed SILAC labeling and proteomics. A) Schematic representation of the pulsed SILAC–based proteomics experiment. B) Overall experimental workflow. R1, R2, and R3 represent triplicate experiments.

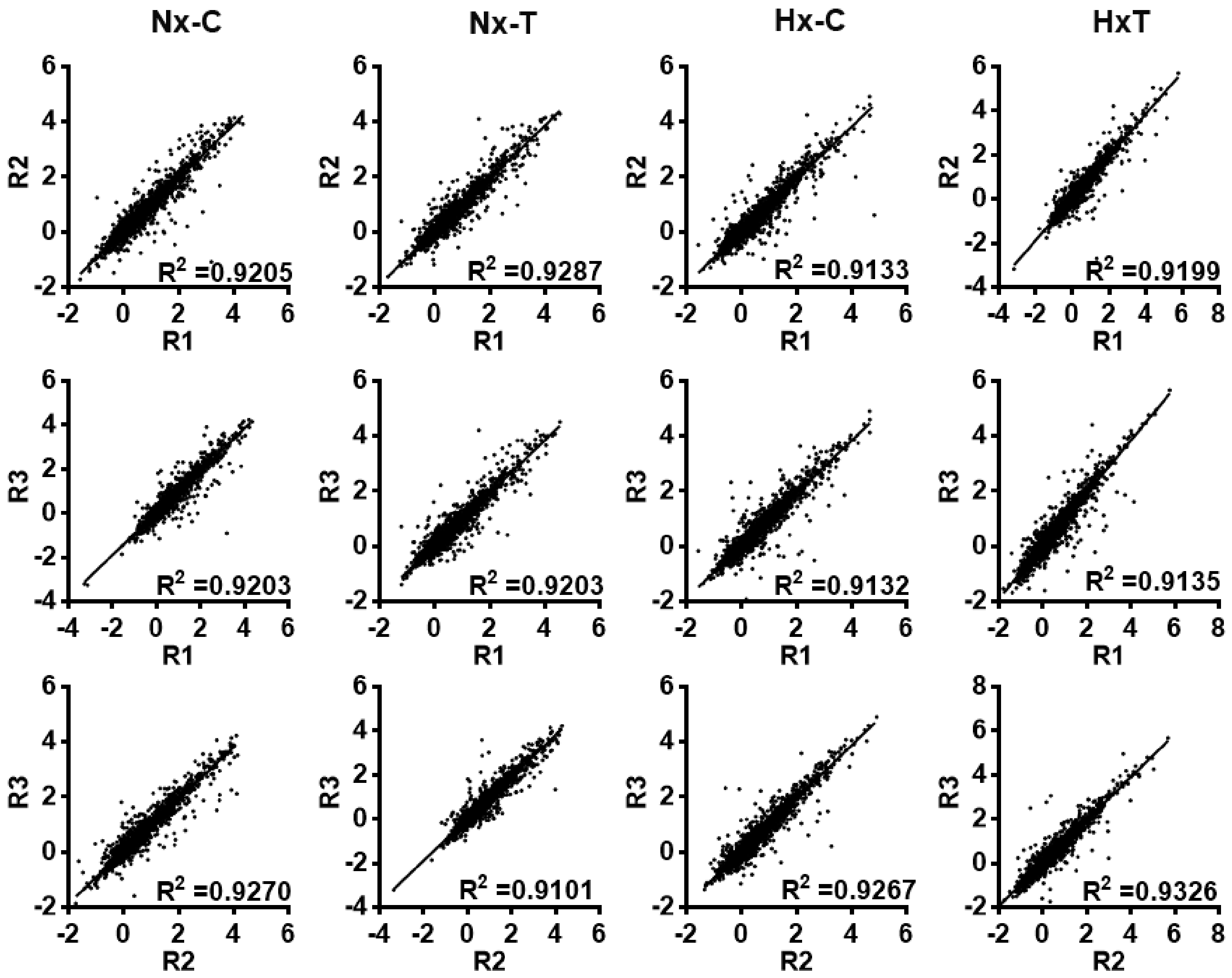

Figure S4: Linear regression plots representing the correlation among triplicate experiments under different experimental conditions. X and Y axes represent the log_2-transformed heavy/light (H/L) SILAC ratio of proteins from respective experimental replicates. R^2 value represents the correlation between replicates, with the high correlations indicating the reliability of the detection of the pulsed SILAC experiment.

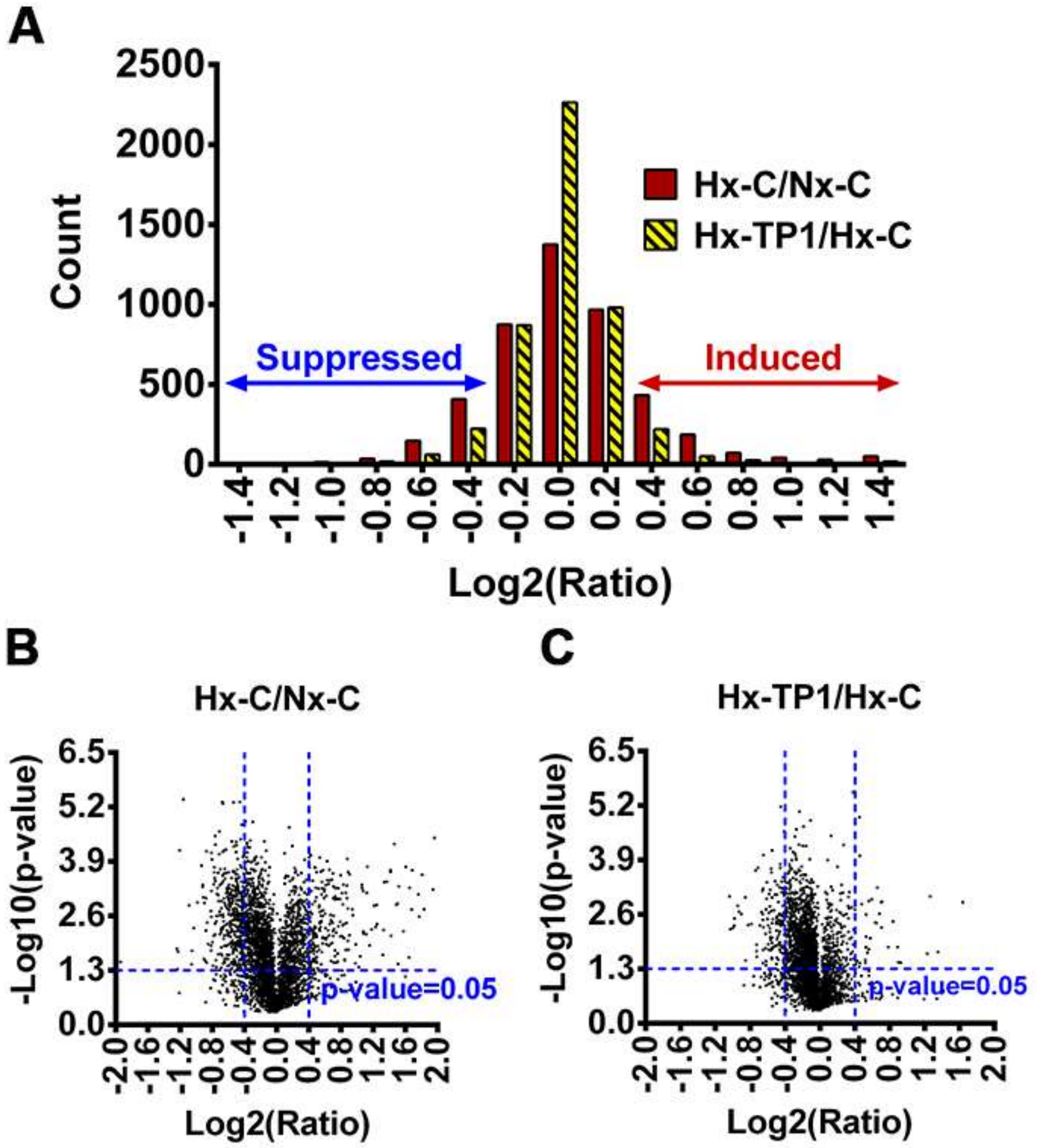

Figure S5: Statistical analysis of proteomic data. A) Frequency distribution plot of fold-change ratio. Cutoff ratio values of <0.76 [log2 (Ratio) < −0.4] and >1.32 [log2 (Ratio) > 0.4] were set for the suppressed and induced proteins, respectively. B, C) Volcano plot highlighting statistically significant differential protein synthesis during hypoxia (ratio of Hx-C/Nx-C) (B) and TP1 treatment during hypoxia (Hx-TP1/Hx-C) (C). HUVEC-CS cells were cultured, SILAC-labeled, and quantified under the respective experimental conditions, including normoxic (Nx-C)/hypoxic (Hx-C) vehicle control and normoxic (Nx-TP1)/hypoxic (Hx-TP1) TP1-treated conditions. Statistical analysis was performed using triplicate experiments. *P < 0.05.

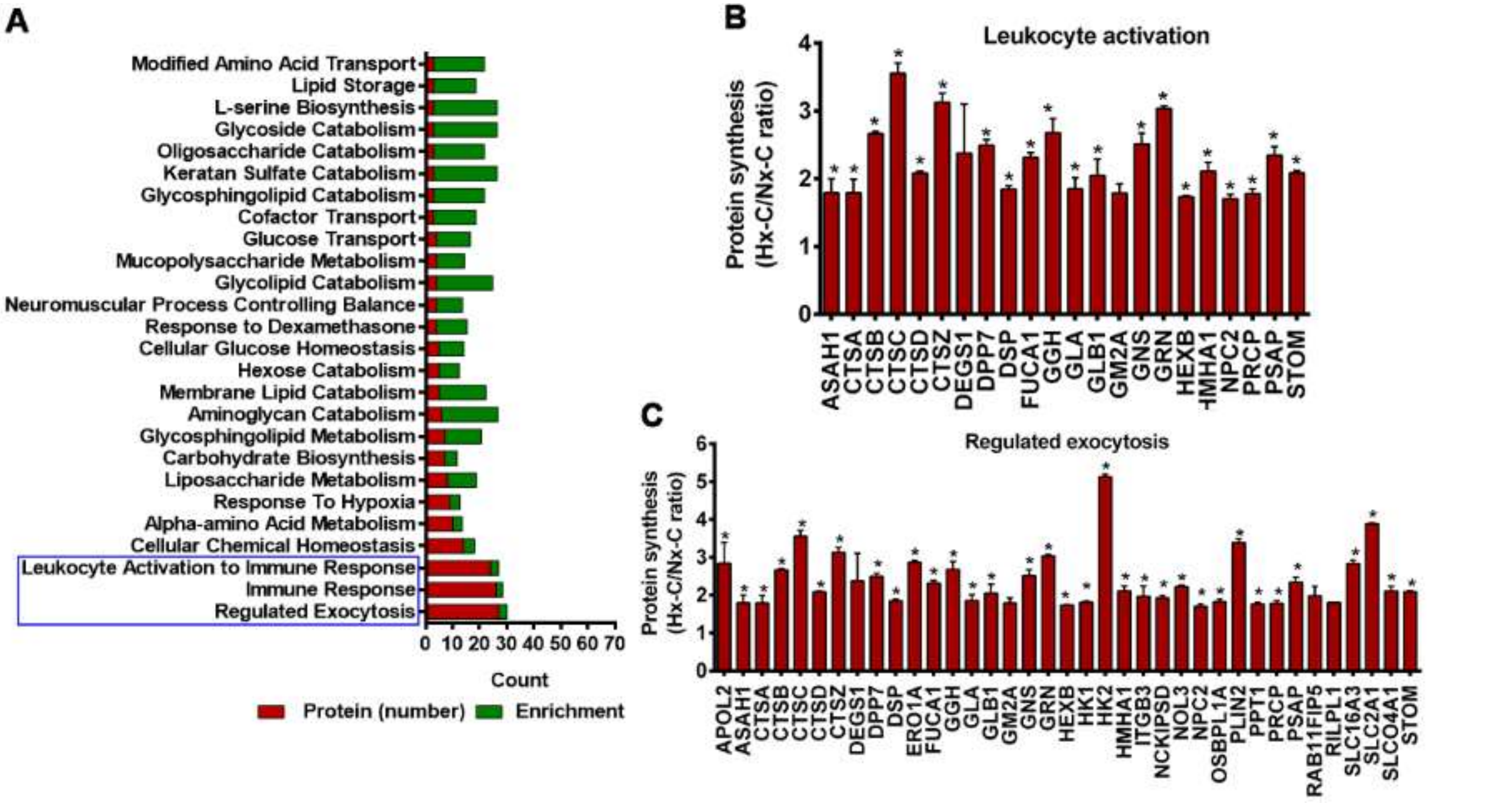

Figure S6: Hypoxia-induced biological processes in endothelial cells (ECs). A) Bioinformatics-based enrichment analysis of the hypoxia-induced biological processes in ECs. B, C) Relative abundance of hypoxia-induced proteins involved in the regulation of bioprocesses, including immune responses and leukocyte activation (B) and exocytosis (C). Quantitation values are means ± standard error of the mean calculated from triplicate experiments. *P < 0.05.

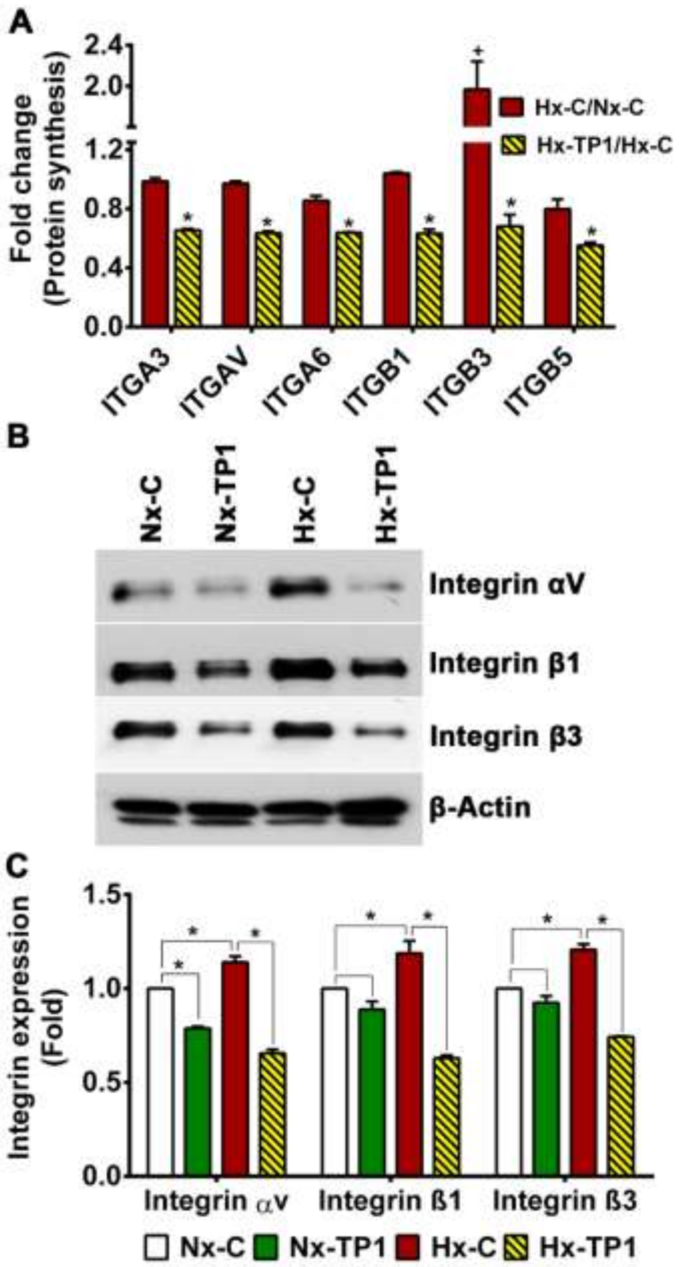

Figure S7: Ginsentide TP1 suppresses integrin expression in hypoxic endothelial cells. A) Relative change in newly synthesized integrin subtypes under the hypoxic condition (Hx-C/Nx-C) and TP1 treatment under the hypoxic condition (Hx-TP1/Hx-C). Significance $^{+}P < 0.05$ vs. Nx-C and $^{*}P < 0.05$ vs. Hx-C. B) Western blot images showing the expression of integrin subtypes under the indicated experimental conditions. C) Graphical representation of the normalized expression of integrin subtypes under the respective experimental conditions. HUVEC-CS cells were cultured and treated with PBS or 20 µM TP1 under normoxic or hypoxic conditions for 24 h. Quantitation values are means ± standard error of the mean, which were calculated using triplicate experiments. $^{*}P < 0.05$.

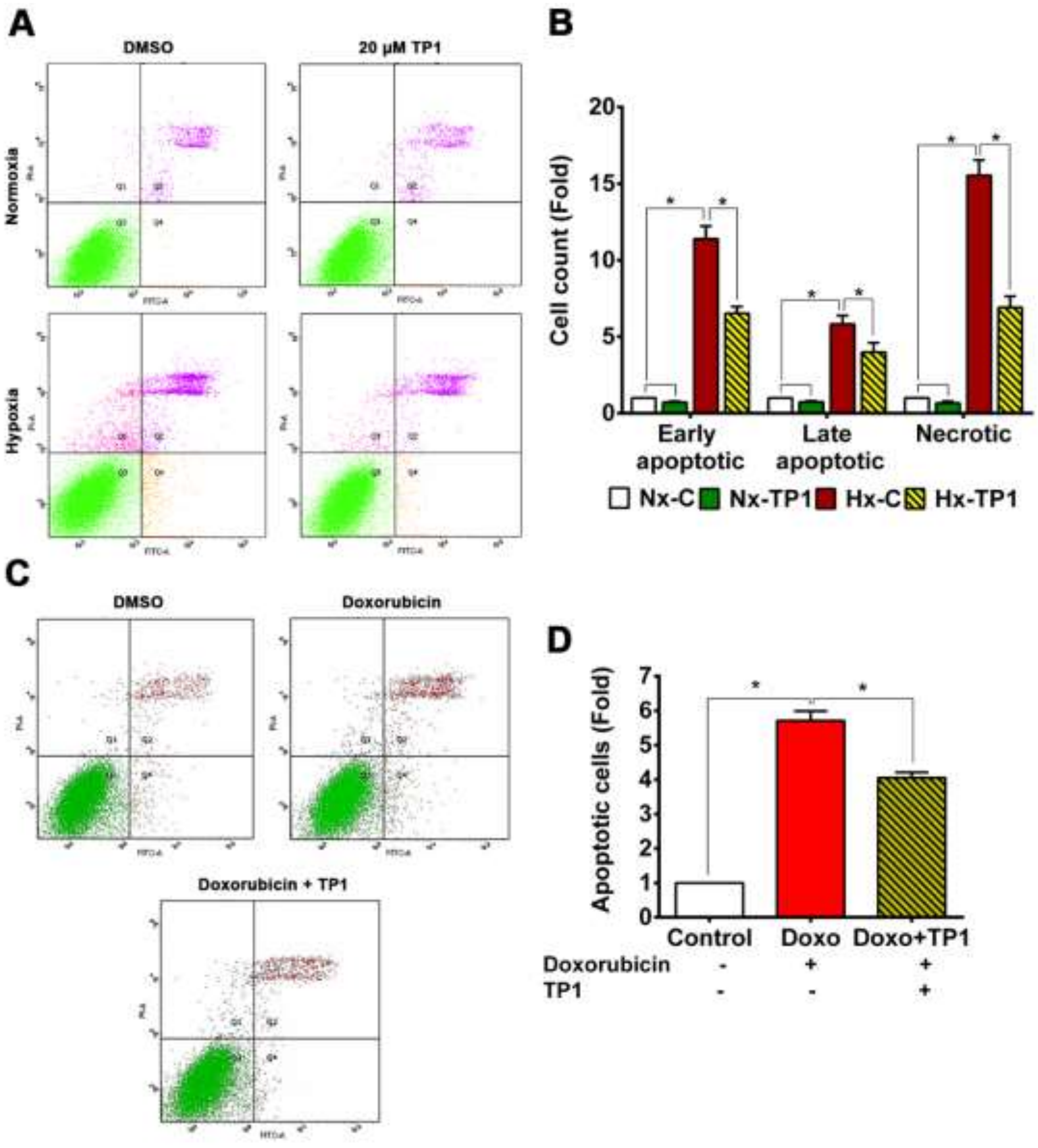

Figure S8: TP1 prevents apoptosis in HUVEC-CS. A) Annexin V and PI staining in HUVEC-CS cells treated with 20 µM TP1 and DMSO at normoxic (Nx-C) or hypoxic (Hx-C) conditions. The third quadrant represents living cells (Annexin V, PI negative), the first one early apoptotic cells (Annexin V positive, PI negative), the second late apoptotic (Annexin and PI positive), and the fourth necrotic or dead cells (Annexin V negative and PI positive). B) Relative quantification was based on cell count. Statistical significance was calculated using three independent experimental replicates. Data are means ± standard error of the mean for triplicate experiments. *P < 0.05. C) Annexin V and PI staining of HUVEC-CS cells treated with 0.2µg/ml doxorubicin with or with 20 µM TP1 co-treatment. DMSO was used as vehicle control. D) Relative quantification of doxorubicin-induced apoptotic cells under respective treatment conditions. Data are means ± standard error of the mean for triplicate experiments. *P < 0.05.

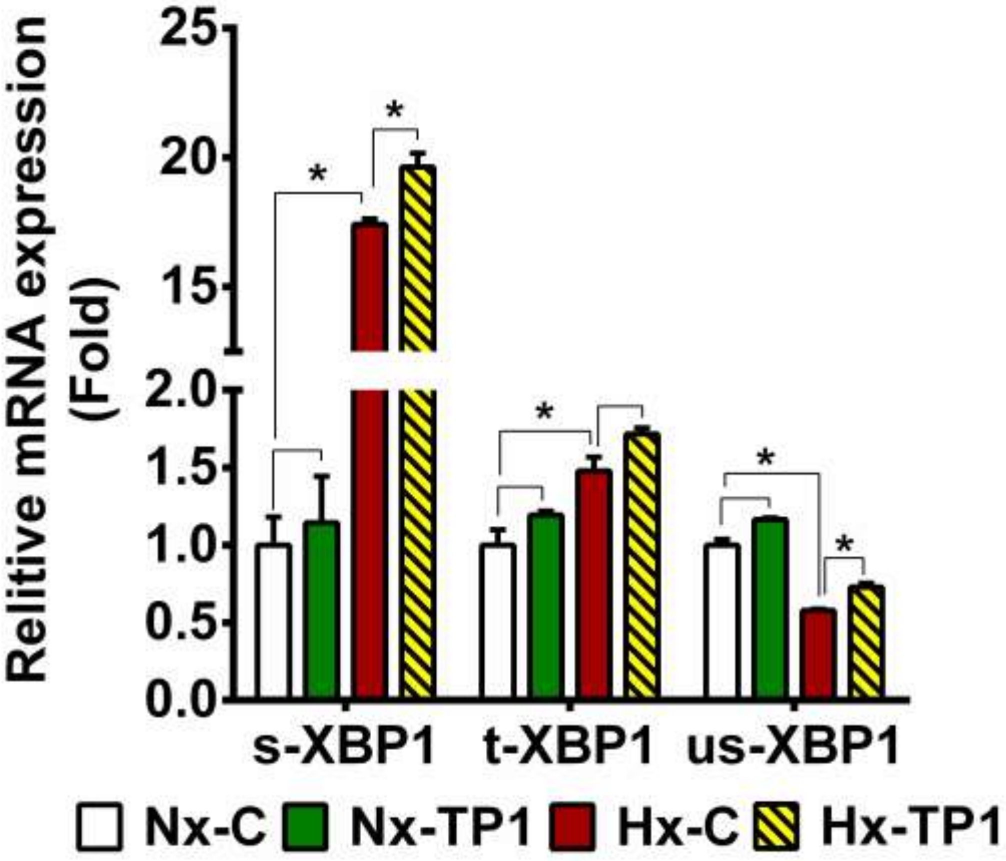

Figure S9: Effect of TP1 on the hypoxia-induced unfolded protein response (UPR) in endothelial cells. Relative mRNA expression levels of the UPR marker gene XBP1, including total XBP (t-XBP1), its spliced isoform (s-XBP1), and its unspliced isoform (us-XBP1), under the indicated experimental conditions. Quantitation values are means with ± standard deviations from triplicate biological replicates. *P < 0.05. HUVEC-CS cells were cultured and treated with PBS or TP1 for 24 h under normoxic or hypoxic conditions. Vehicle control normoxic (Nx-C) and hypoxic (Hx-C) and TP1-treated normoxic (Nx-TP1) and hypoxic (Hx-TP1) treatments are shown.

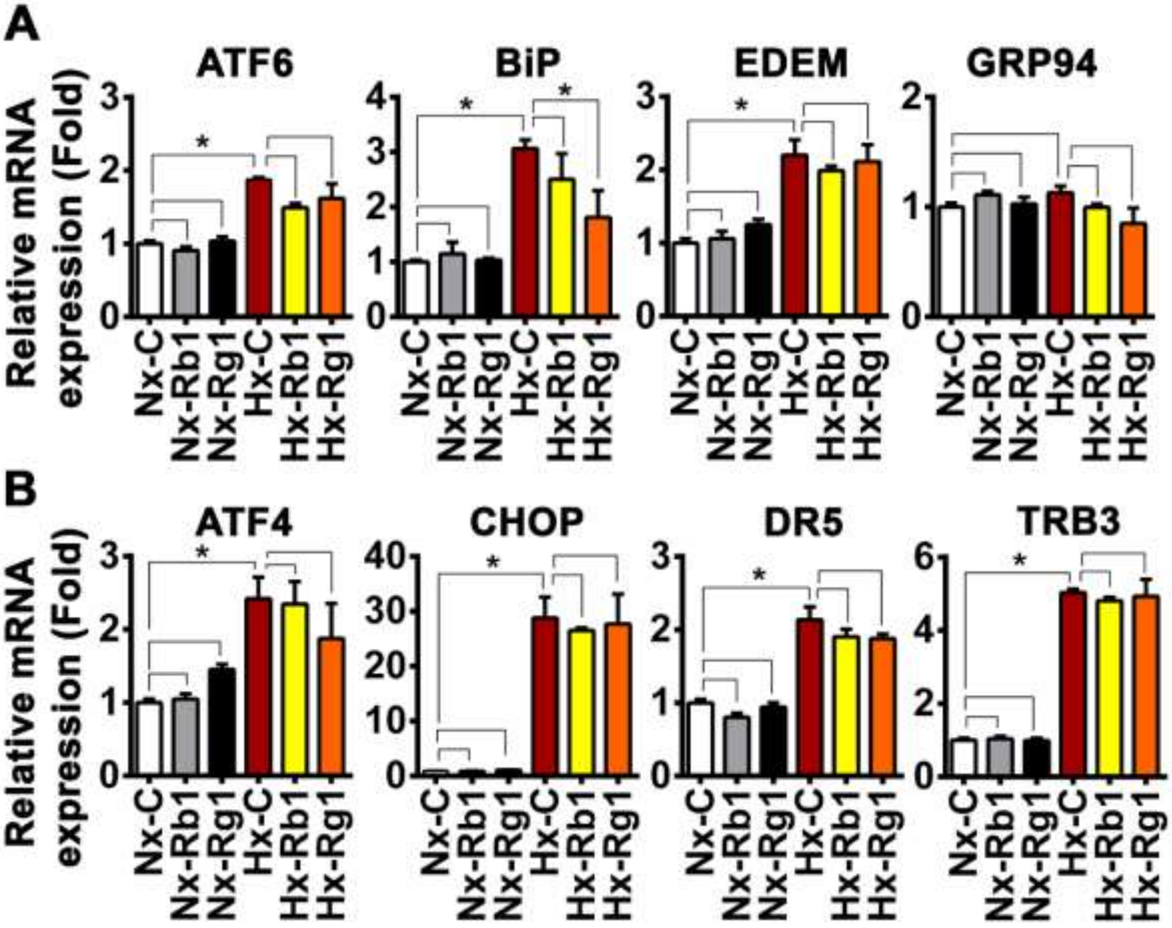

Figure S10: Effects of ginsenosides on hypoxia-induced ER stress–related gene expression in ECs. A, B) Relative mRNA expression levels of ER stress–related adaptive unfolded protein response (UPR) transduction cascade genes (A) and UPR-mediated death signaling cascade genes (B) in HUVEC-CS cells cultured under the indicated experimental conditions. Mean ± standard error of the mean were calculated from triplicate biological replicates. Vehicle control cells were cultured with PBS for 24 h under normoxic (Nx-C) or hypoxic (Hx-C) conditions, and 20 µM ginsenoside Rb1- or Rg1-treated cells were cultured for 24 h under normoxic (Nx-Rb1/Nx-Rg1) or hypoxic (Hx-Rb1/Hx-Rg1) conditions. *$P < 0.05$.

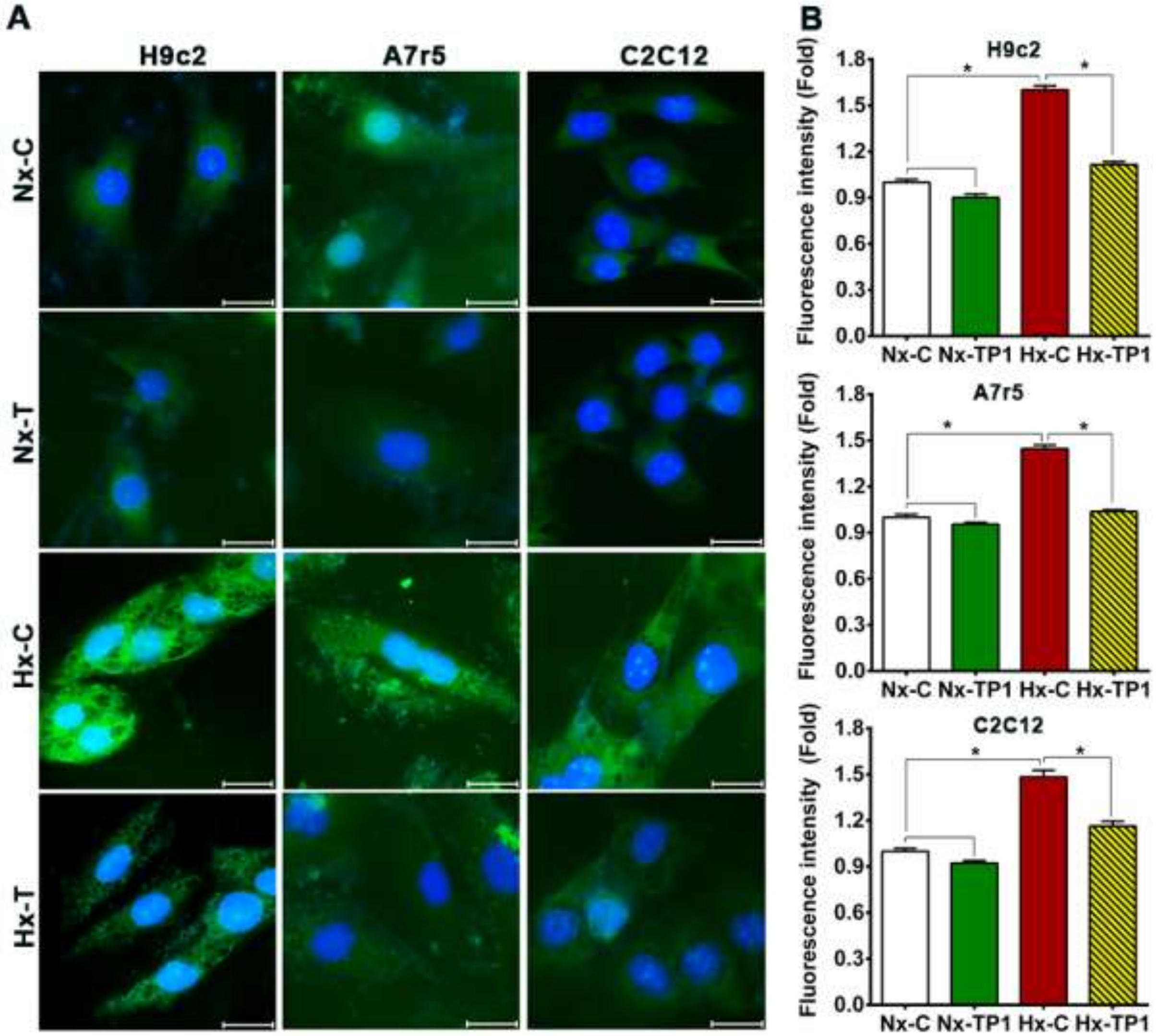

Figure S11. TP1-mediated recovery of ER/protein homeostasis in hypoxic cardiovascular cell types. A) Thioflavin-T (ThT) staining of aggregated proteins in the cardiovascular cell lines H9c2, A7r5, and C2C12 cultured under various experimental conditions. Protein accumulation was visualized by staining with ThT (green), and nuclei were counterstained with DAPI (blue). Scale bar: 20 µm. B) Relative quantification of protein accumulation under different experimental conditions was performed by calculating the average fluorescence intensity of >100 individual cells from four experimental replicates. Vehicle control cells were cultured for 24 h under normoxic (Nx-C) or hypoxic (Hx-C) conditions and TP1-treated cells were cultured for 24 h under normoxic (Nx-TP1) or hypoxic (Hx-TP1) conditions. *P < 0.05.

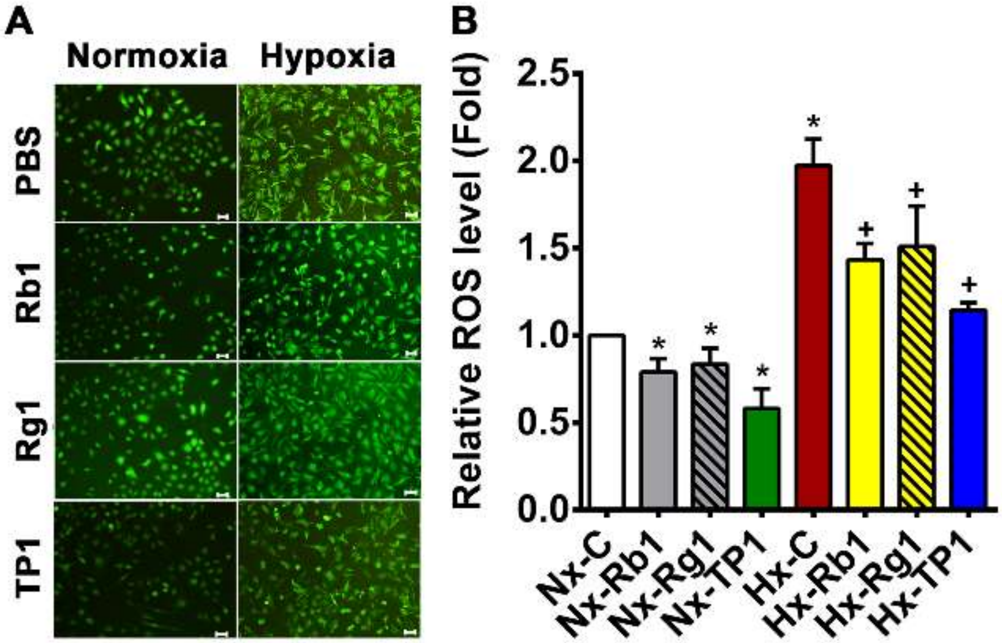

Figure S12: Effect of ginsenoside treatment on the intracellular ROS load in ECs. A) Effect of Rb1 and Rg1 on intracellular ROS levels in HUVEC-CS cells cultured under the indicated experimental conditions. Scale bar: 25 µm. B) Relative quantification was performed using relative fluorescence intensity, and statistical significance was calculated using three biological replicates. Data are means ± standard error or the mean. Normoxic or hypoxic HUVEC-CS cells were treated with PBS (vehicle control), 20 µm ginsenoside Rb1 or Rg1, or 20 µm ginsentide TP1 (positive control) for 24 h. $^*P < 0.05$.

Full blot images. Western blot analysis of HUVEC-CS endothelial cells on 20µM ginsentide TP1 treatment under normoxic and hypoxic conditions. Vehicle control cells were cultured for 24h under normoxia (Nx-C) or hypoxia (Hx-C) conditions. Cells were treated with TP1 for 24h under normoxia (Nx-TP1) or hypoxia (Hx-TP1)

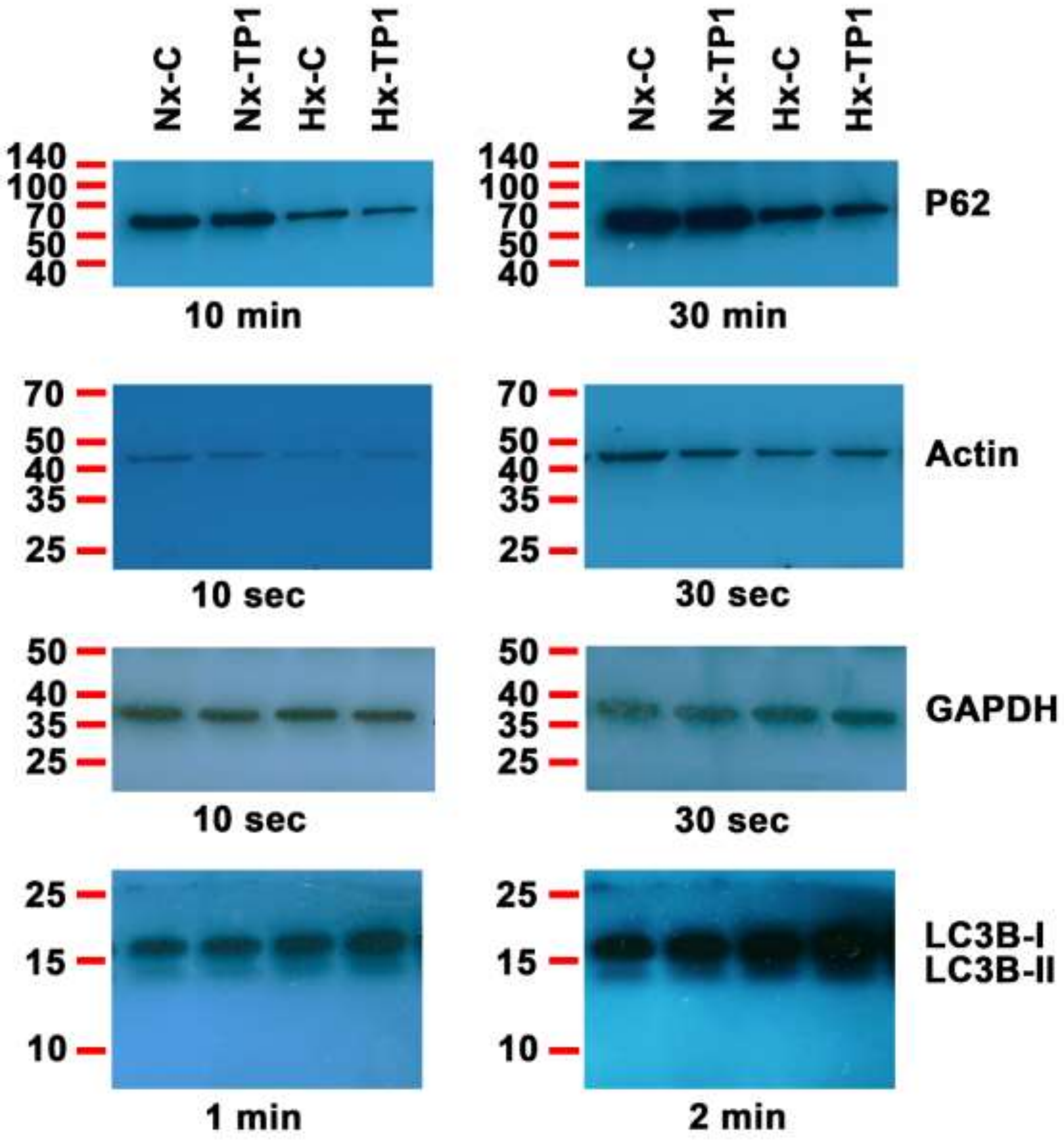

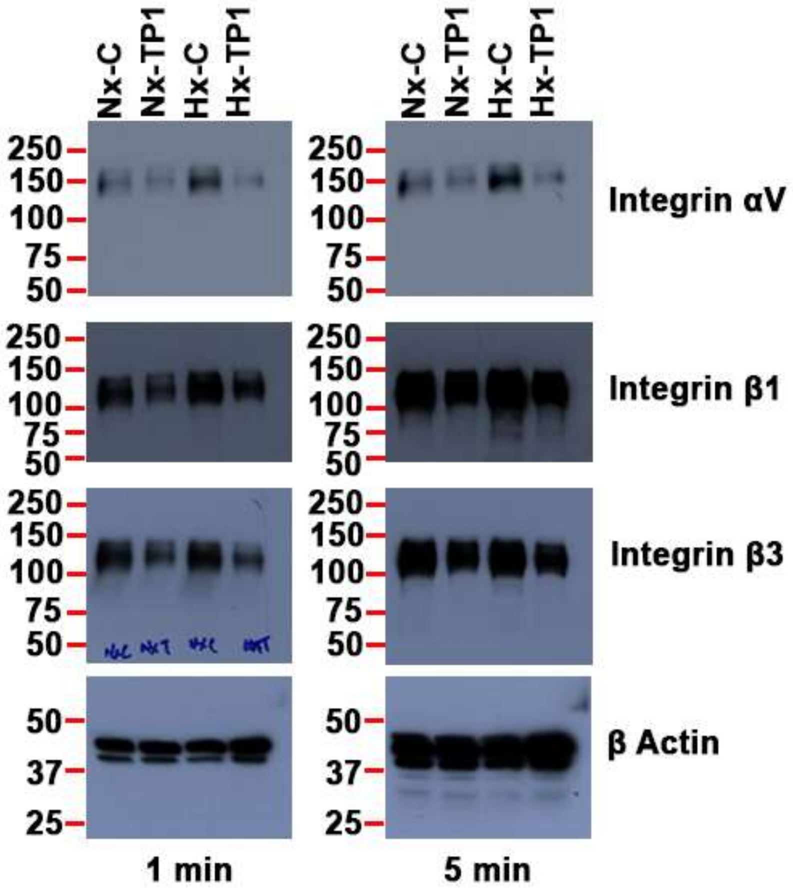
Nx-C
Nx-TP1
Hx-C
Hx-TP1
250
150
100
75
50
Integrin αV
Integrin β1
Integrin β3
37
25
β Actin
1 min
5 min

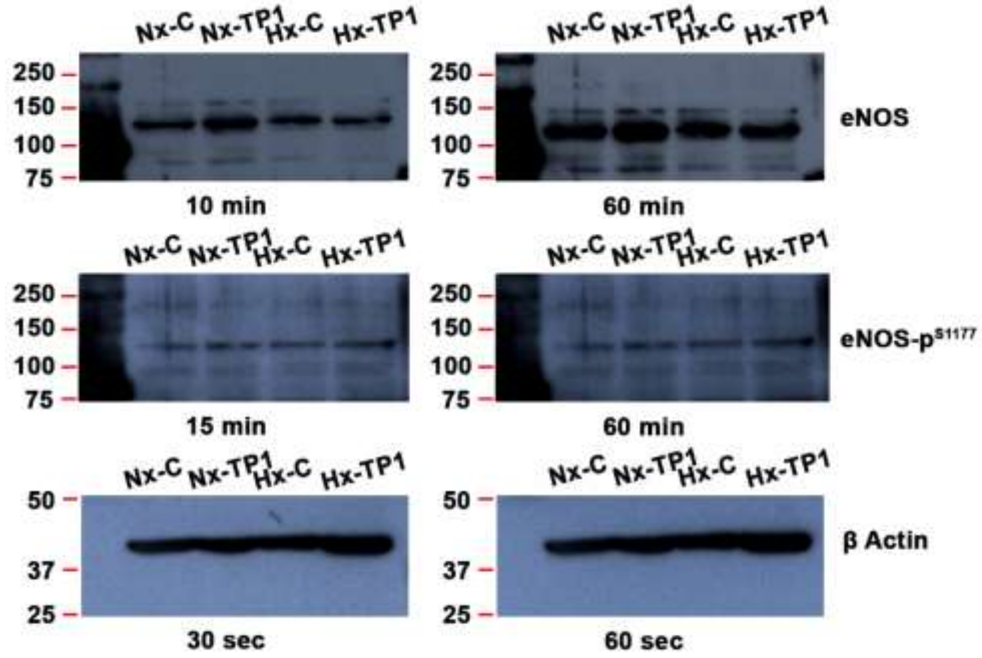
Nx-C
Nx-TP1
Hx-C
Hx-TP1
250
150
100
75
eNOS
10 min
60 min
eNOS-p^{S1177}
15 min
60 min
50
37
25
β Actin
30 sec
60 sec

Supplementary Tables

Table S1: List of genes and corresponding primers used in quantitative RT-PCR.

Genes	Forward primer sequence (5´–3´)	Reverse primer sequence (5´–3´)
**ALCAM**	CGCAATGCAACAGGAGACTA	GGCTAGATCGAAGCCTGATG
**ICAM1**	GGCTGGAGCTGTTTGAGAAC	ACTGTGGGGTTCAACCTCTG
**L1CAM**	GCCAAAGGAGACAGTGAAGC	GCGTGGCAGATGTAGTCTGA
**VCAM1**	CAGACAGGAAGTCCCTGGAA	TTCTTGCAGCTTTGTGGATG
**ATF4**	GTTCTCCAGCGACAAGGCTA	ATCCTGCTTGCTGTTGTTGG
**CHOP**	AGAACCAGGAAACGGAAACAGA	TCTCCTTCATGCGCTGCTTT
**DR5**	CACCAGGTGTGATTCAGGTG	CCCCACTGTGCTTTGTACCT
**TRB3**	TGGTACCCAGCTCCTCTACG	TTCTCCAGCACCAGCTTCTT
**ATF6**	GCCTTTATTGCTTCCAGCAG	TGAGACAGCAAAACCGTCTG
**BiP**	TGTTCAACCAATTATCAGCAAACT	TTCTGCTGTATCCTCTTCACCAGT
**EDEM**	CAAGTGTGGGTACGCCACG	AAAGAAGCTCTCCATCCGGTC
**GRP94**	GAAACGGATGCCTGGTGG	GCCCCTTCTTCCTGGGTC
**sXBP1**	CTGAGTCCGAATCAGGTGCAG	ATCCATGGGGAGATGTTCTGG
**usXBP1**	CAGCACTCAGACTACGTGCA	ATCCATGGGGAGATGTTCTGG
**Total-XBP1**	TGGCCGGGTCTGCTGAGTCCG	ATCCATGGGGAGATGTTCTGG
**RPLP0**	TCGACAATGGCAGCATCTAC	GCCTTGACCTTTTCAGCAAG
**RPL13A**	GTACGCTGTGAAGGCATCAA	CGCTTTTTCTTGTCGTAGGG
**B-actin**	AGAGCTACGAGCTGCCTGAC	AGCACTGTGTTGGCGTACAG
**18S**	GTAACCCGTTGAACCCCATT	CCATCCAATCGGTAGTAGCG

sXBP1: Spliced XBP1 and usXBP1: Unspliced XBP1
